# Supplementary figures and images for: Immunophenotype and antitumor activity of cytokine-induced killer cells from patients with hepatocellular carcinoma
Source: PLoS One. 2023 Jan 4;18(1):e0280023. doi: 10.1371/journal.pone.0280023 (PMC9812323; doi:10.1371/journal.pone.0280023)

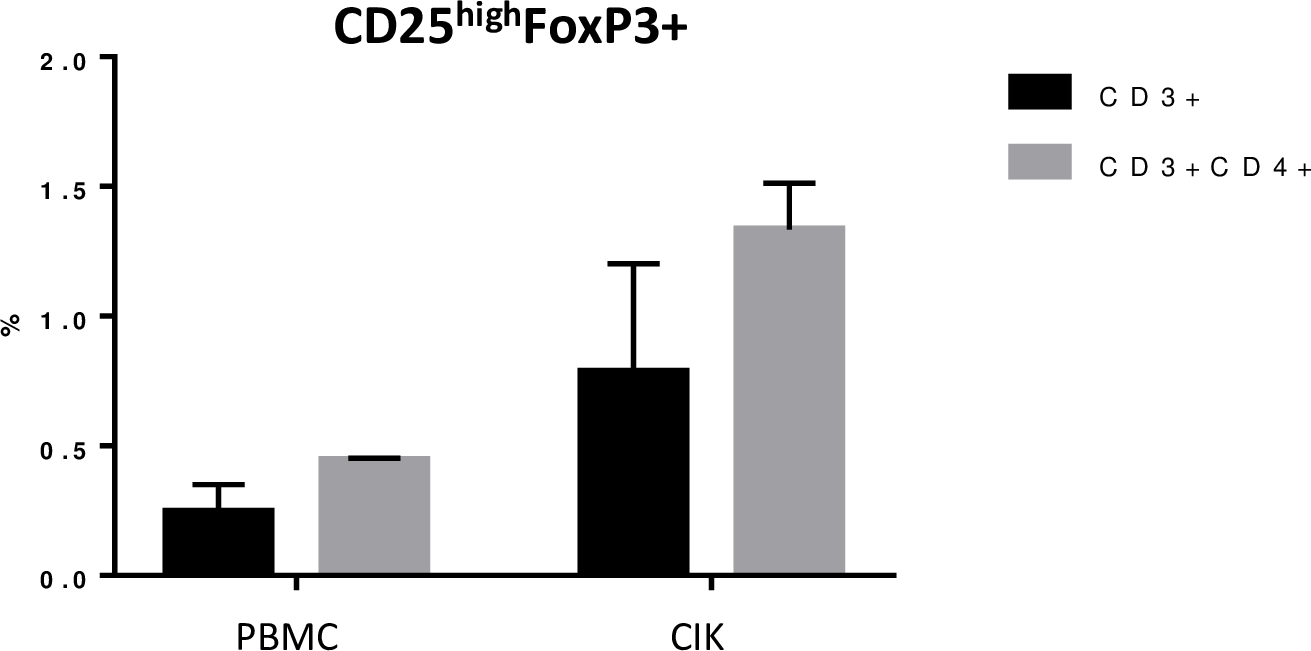

Supplement: S1 Fig — Expression of CD25highFoxP3+ T cells were low on CIK cells and PBMCs (N = 3). (TIF) [file pone.0280023.s001.tif]

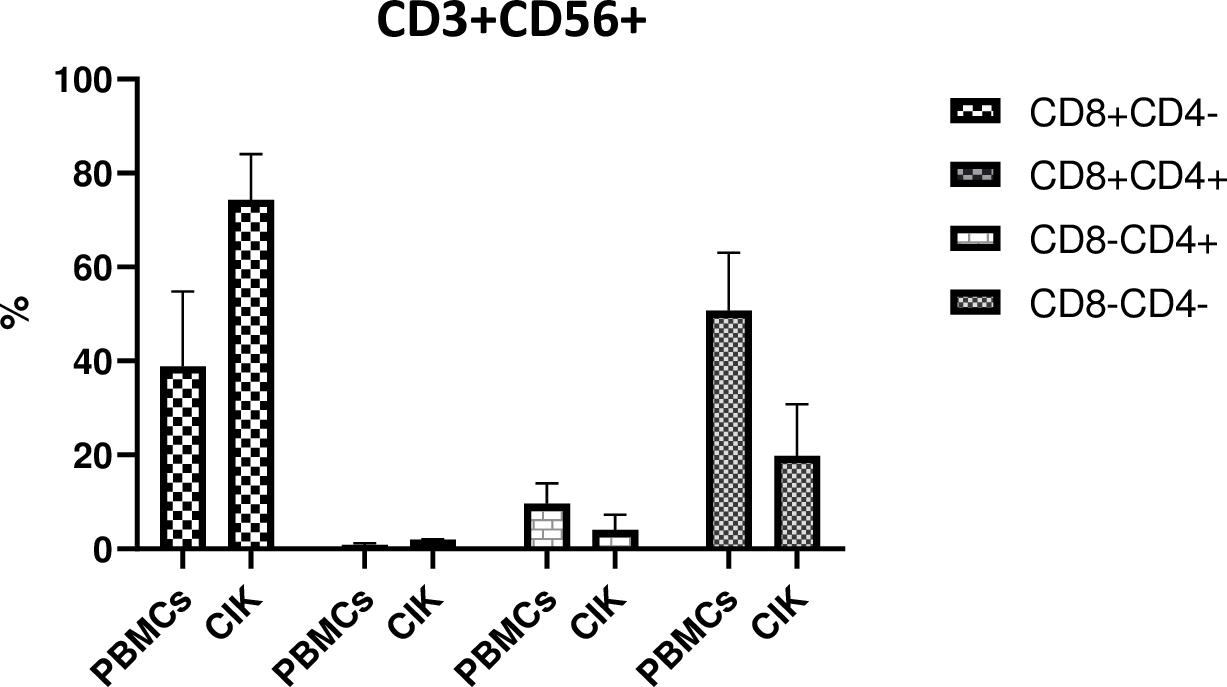

Supplement: S2 Fig — CD56 was major expressed on CD3+CD8+ T cells in CIK cells (N = 5). (TIF) [file pone.0280023.s002.tif]

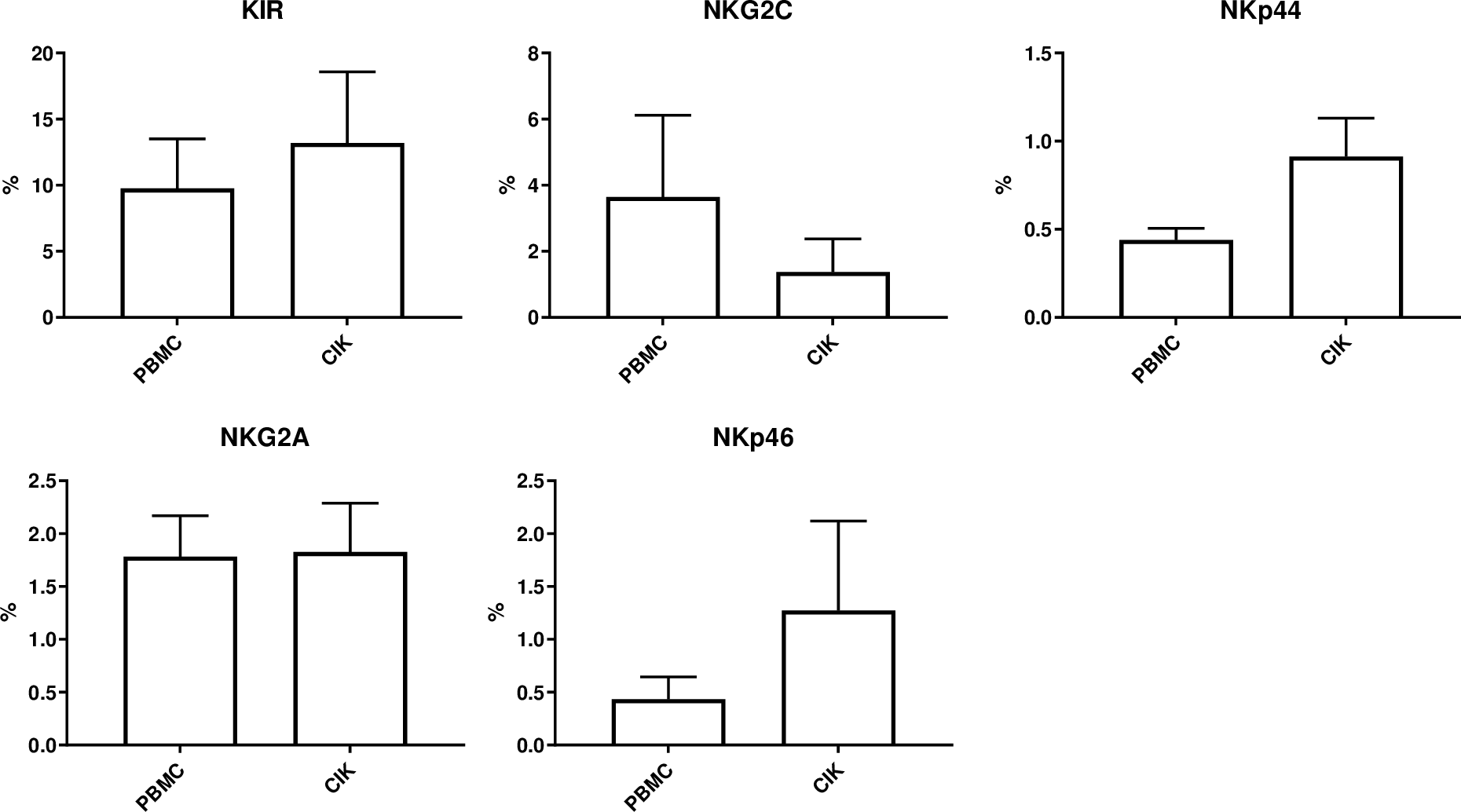

Supplement: S3 Fig — Expression of NKG2C, NKG2A, KIR, NKp46 and NKp44 were low on CIK cells and PBMCs (N = 5). (TIF) [file pone.0280023.s003.tif]

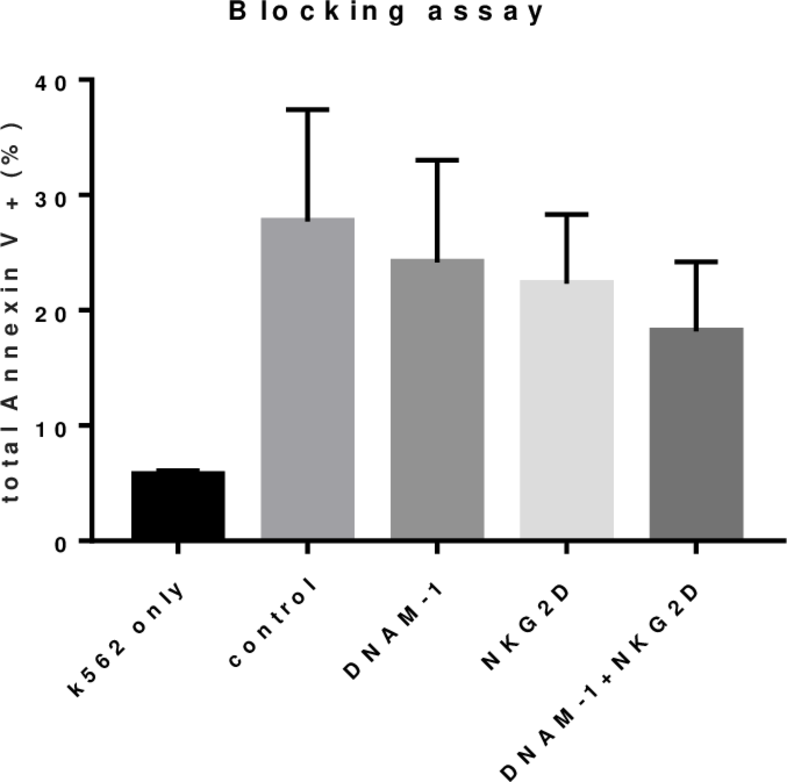

Supplement: S4 Fig — The blocking assay was performed to explore the potential mechanism for the cytolytic activity of CIK cells from HCC patients via a DNAM-1 and NKG2D-dependent manner (N = 2). The synergic use of anti-DNAM-1 and anti-NKG2D antibodies decreased the cytolytic ability of CIK cells obtained from HCC patients. (TIF) [file pone.0280023.s004.tif]
