# Supplementary material for: Immunophenotype and antitumor activity of cytokine-induced killer cells from patients with hepatocellular carcinoma
Source: PLoS One. 2023 Jan 4;18(1):e0280023. doi: 10.1371/journal.pone.0280023 (PMC9812323; doi:10.1371/journal.pone.0280023)
Supplement: Attachment — Submitted filename: Response to reviewers.docx [file pone.0280023.s005.docx]

**Point-by-point response to Journal requirements and Reviewer’s comments (**PONE-S-22-22620**)**

(>>: our responses)

(Modifications and the inclusion of new data have been marked with tracked changes in revised manuscript uploaded as the “Revised Manuscript with Tracked Changes” and summarized in List of amendments to manuscript, page 6)

Journal Requirements:

1. Please ensure that your manuscript meets PLOS ONE's style requirements, including those for file naming.
>>: Our manuscript had modified to meet PLOS ONE's style requirements.

2. To comply with PLOS ONE submissions requirements, in your Methods section, please provide additional information regarding the experiments involving animals and ensure you have included details on (1) methods of sacrifice, (2) methods of anesthesia and/or analgesia, and (3) efforts to alleviate suffering.
>>: Thanks for your kindly reminder. We had modified the Methods and add a paragraph in the “Ethics statement” to clearly describe the methods of sacrifice, anesthesia and efforts to alleviate suffering.

3. Please provide additional details regarding participant consent. In the ethics statement in the Methods and online submission information, please ensure that you have specified (1) whether consent was informed and (2) what type you obtained (for instance, written or verbal, and if verbal, how it was documented and witnessed). If your study included minors, state whether you obtained consent from parents or guardians. If the need for consent was waived by the ethics committee, please include this information.
>>: The inform consents were approved by the Institutional Review Board of Chang Gung Medical Foundation and obtained by written, which had been described in the “Ethics statement”.

4. Thank you for stating in your Funding Statement:
"This work was supported by CMRPG3F1301, CMRPG3J0871, CMRPG5J0101 and CMRPG5K0221 from Medical Research Project Fund, Chang Gung Memorial Hospital, Taiwan and MOST 107-2314-B-182A-157 from Ministry of Science and Technology, Taiwan."

Please provide an amended statement that declares *all* the funding or sources of support (whether external or internal to your organization) received during this study, as detailed online in our guide for authors at http://journals.plos.org/plosone/s/submit-now.  Please also include the statement “There was no additional external funding received for this study.” in your updated Funding Statement. Please include your amended Funding Statement within your cover letter. We will change the online submission form on your behalf.
>>: Thanks again for your reminder. We had revised the “Acknowledgements” and described the amended Funding Statement in our cover letter.

5. Thank you for stating the following financial disclosure: 
"This work was supported by CMRPG3F1301, CMRPG3J0871, CMRPG5J0101 and CMRPG5K0221 from Medical Research Project Fund, Chang Gung Memorial Hospital, Taiwan and MOST 107-2314-B-182A-157 from Ministry of Science and Technology, Taiwan."

Please state what role the funders took in the study. If the funders had no role, please state: ""The funders had no role in study design, data collection and analysis, decision to publish, or preparation of the manuscript."" 
If this statement is not correct you must amend it as needed. Please include this amended Role of Funder statement in your cover letter; we will change the online submission form on your behalf.
>>: We had revised the “Acknowledgements” and described the amended Funding Statement in our cover letter.

6. PLOS requires an ORCID iD for the corresponding author in Editorial Manager on papers submitted after December 6th, 2016. Please ensure that you have an ORCID iD and that it is validated in Editorial Manager. To do this, go to ‘Update my Information’ (in the upper left-hand corner of the main menu), and click on the Fetch/Validate link next to the ORCID field. This will take you to the ORCID site and allow you to create a new iD or authenticate a pre-existing iD in Editorial Manager. Please see the following video for instructions on linking an ORCID iD to your Editorial Manager account: https://www.youtube.com/watch?v=_xcclfuvtxQ
>>: I had updated my ORCID iD.

7. Please upload a new copy of Figure 4 as the detail is not clear. Please follow the link for more information: https://blogs.plos.org/plos/2019/06/looking-good-tips-for-creating-your-plos-figures-graphics/"" https://blogs.plos.org/plos/2019/06/looking-good-tips-for-creating-your-plos-figures-graphics/
>>: I had uploaded a new clear Figure 4. Clear Figures 2 and 3 were also uploaded.

Reviewers' comments:

Reviewer #1: Major Points:
Please elaborate what is new finding in this paper?
>>: In this article, we demonstrated the intravenous human CIK cells could infiltrate to tumor site and contain anti-tumor ability in xenograft mouse model. To our knowledge, this finding had not proved in other papers. This study further supports the possible mechanism of the killing effects of CIK cells as a group of highly activated, inflamed site-trafficking bystander T cells.

Could you correlate expression of DNAM or NKG2D on K562 and HepG2 with the susceptibility to killing by CIK?
>>: We found that synergic use of anti-DNAM-1 and anti-NKG2D antibodies decreased the cytolytic ability of CIK cells obtained from HCC patients, suggesting that CIK cells may display cytotoxicity in a DNAM-1 and NKG2D-dependent manner. We had added the description in Results and S4 Fig. Thanks for your suggestions.

CIK from how many HCC patients were tested?
>>: Total 10 HCC patients were tested for culture of CIK cells in this research. Among them, the CIK from 5 patients were studied for the immunophenotype and the ex-vivo killing ability. The CIK from other 5 patients were tested for in vivo NOD/SCID mice model. These had been described in the Results.

How are you defining CIK here; CD3+CD4+ T cells, CD3+CD8+ T cells or both?
>>: CIK cells are defined as generation by ex vivo incubation of PBMC with cytokines. After cytokine stimulation, CIK cells could generate a group of immune effector cells featuring a mixed T- and NK cell-like phenotype, including both CD3+CD4+ and CD3+CD8+ T cells. However, the percentage of CD3+CD8+ cells increased after expansion (CIK cells vs. PBMCs: 72% vs. 21%, p < 0.05). The percentage of CD3+CD4+ cells decreased after expansion (CIK cells vs. PBMCs: 22% vs. 73%, p < 0.05) (Fig 2B).

Is CD56 expressed on CD3+CD4+ T cells or only on CD3+CD8+ T cells?
>>: In CIK cells, CD56 was major expressed on CD3+CD8+ T cells. Among CD3+CD56+ T cells, CD8+CD4- T cells were 74.3%, but CD8-CD4+ T cells was only 4%. We had added the description into Results and S2 Fig.

What was the % of CD25^high^FoxP3+ T cells in expanded cells?
>>: CD25^high^FoxP3+ T cells was only 1.3% in CIK cells. We had added the description into Results and S1 Fig.

What would be the effects on CIK, if IL-15 is added to expand and induce CIK?
>>: Rettinger E et al. had studies the effect of IL-15 for CIK cells in models of human leukemia xenografts (Rettinger E et al. Bone Marrow Transplant. 2013). In vitro cytotoxicity of IL-15 activated CIK cells was significantly enhanced compared to IL-2 activated controls and in vivo anti-leukemic efficacy, showed homing of IL-15 activated CIK cells to leukemia sites, leukemia control, and when repeatedly given complete disease clearance indicated by improved survival. In our recent research, we are going on studying to modify the cytokine protocol, including IL-15 and other else. Currently, we found the memory and innate T cells would increase after stimulation by multiple cytokines including IL-15. We add the description and reference of IL-15 function for expansion and function of CIKs to “Discussion”. Thank you for this important information.

Why did not you investigate expression of other NK cell receptors like NKG2C, NKG2A, KIR, NKp46, etc?
>>: Expression of these NK receptors including NKG2C, NKG2A, KIR, NKp46 and NKp44 were all low in both CIK cells and PBMCs. We had added the information into Results and S3 Fig. Thank you for this important suggestion.

Minor points:
Page 11: The results of other treatments for palliative purpose remain……(not remains).
>>: We had corrected “remains” to “remain”

Page 13: A renowned worldwide cancer treatment guideline has adopted………(provide ref).
>>: We add the reference in this sentence. Thanks for the reminder.

**List of amendments to manuscript (Manuscript Number:** PONE-S-22-22620**)**

Reviewer’s comments have been addressed in detail in the “**Point-by-point response to reviewer’s comments**”. Modifications and the inclusion of new data have been marked in red in the revised manuscript and summarized by the following points:

**Introduction**

P5: “remains” was changed to “remain”.

P7: A reference [26] was cited.

**Materials and methods:**

P8-P9: Add a paragraph: “Ethics statement”.

P11: Add a paragraph: “Blocking assay”.

**Results:**

P14, 1^st^ paragraph: Add description for total patient number in this study.

P14, 2^nd^ paragraph: Add description about expression of CD25^high^FoxP3+ T cells and CD56 on CD3+CD4+ and CD3+CD8+ T cells.

P15: Explain for the expression of NKG2C, NKG2A, KIR, NKp46 and NKp44.

P16: Explain the cytolytic ability of CIK cells by a DNAM-1 and NKG2D-dependent manner.

**Discussion:**

P23: Add description and references of IL-15 function for expansion and function of CIK cells.

**Acknowledgements:**

P24: Remove the funding statement from Acknowledgement, and explain in our cover letter.

**References:**

P27: Add a new reference [26]. The order of references was modified accordingly.

P30: Add a new reference [52].

**Supporting information:**

S1-S4 Figs: new figures were added.

P34: Figure legends were added for S1-4 Figs.
