# Supplementary material for: Immunophenotype and antitumor activity of cytokine-induced killer cells from patients with hepatocellular carcinoma
Source: PLoS One. 2023 Jan 4;18(1):e0280023. doi: 10.1371/journal.pone.0280023 (PMC9812323; doi:10.1371/journal.pone.0280023)
Supplement: Attachment — Submitted filename: PONE-D-22-17512R1.doc [file pone.0280023.s006.doc]

**Manuscript ID: PONE-D-22-17512R1**

**Manuscript Title: Immunophenotype and antitumor activity of cytokine-induced killer cells from patients with hepatocellular carcinoma**

The authors activated human cytokine-induced killer (CIK) cells ex-vivo and expanded from PBMCs from hepatocellular carcinoma (HCC) patients. They evaluated immunophenotyping and the ex-vivo killing ability of human CIK cells. Furthermore, they intravenously injected human CIK cells into NOD/SCID mice to evaluate the in vivo anticancer ability. They used human HCC cell line, J7, bearing NOD/SCID mice model in this study. This is an important subject and well written manuscript but there are several points should be addressed before its publication.

Non-obese-diabetic–severe combined immunodeficient mice (NOD–SCID) which were reported to lack innate immunity but are limited owing to a residual appearance of T and B cells and remaining natural killer (NK) cells (Cancer Res. 76, 6153–6158 (2016)). Recombination activating gene (RAG)-knockout mice overcome the residual appearance of adaptive immune cells but still show NK cells (Cancer Res. 76, 6153–6158 (2016)). NSG (NOD/SCID-IL2g-/−) strain mice, produced by the targeted mutation of the IL-2 receptor g-chain locus in a previously bred NOD–SCID strain, lack T cells, B cells, macrophages, NK cells and natural killer T cells (NKT) and have become an increasingly used platform for xenograft models (Cancer Res. 76, 6153–6158 (2016); Cancer Lett. 344, 13–19 (2014)). Therefore, we cannot tell the origin of antitumor effect in this study. The authors are better to use RAG-knockout mice or NSG (NOD/SCID-IL2g-/−) mice model in this study.

Another point to be addressed here is that human CIK cells could be affected by immunosuppressive cells (J Hepatol. 2019 Mar;70(3):449-457). Therefore, we need human immunosuppressive cells in murine HCC models to tell the exact anticancer effect of human CIK cells in vivo system.
