# Supplementary material for: Immunophenotype and antitumor activity of cytokine-induced killer cells from patients with hepatocellular carcinoma
Source: PLoS One. 2023 Jan 4;18(1):e0280023. doi: 10.1371/journal.pone.0280023 (PMC9812323; doi:10.1371/journal.pone.0280023)
Supplement: Attachment — Submitted filename: Response to reviewers.docx [file pone.0280023.s007.docx]

**Point-by-point response to Journal requirements and Reviewer’s comments (PONE-D-22-17512R1)**

(>>: our responses)

(Modifications have been marked with tracked changes in revised manuscript uploaded as the “Revised Manuscript with Tracked Changes” and summarized in List of amendments to manuscript, page 4)

Reviewers' comments:

Reviewer #2: Major Points:
The authors activated human cytokine-induced killer (CIK) cells ex-vivo and expanded from PBMCs from hepatocellular carcinoma (HCC) patients. They evaluated immunophenotyping and the ex-vivo killing ability of human CIK cells. Furthermore, they intravenously injected human CIK cells into NOD/SCID mice to evaluate the in vivo anticancer ability. They used human HCC cell line, J7, bearing NOD/SCID mice model in this study. This is an important subject and well written manuscript but there are several points should be addressed before its publication.

Non-obese-diabetic–severe combined immunodeficient mice (NOD–SCID) which were reported to lack innate immunity but are limited owing to a residual appearance of T and B cells and remaining natural killer (NK) cells (Cancer Res. 76, 6153–6158 (2016)). Recombination activating gene (RAG)-knockout mice overcome the residual appearance of adaptive immune cells but still show NK cells (Cancer Res. 76, 6153–6158 (2016)). NSG (NOD/SCID-IL2g-/−) strain mice, produced by the targeted mutation of the IL-2 receptor g-chain locus in a previously bred NOD–SCID strain, lack T cells, B cells, macrophages, NK cells and natural killer T cells (NKT) and have become an increasingly used platform for xenograft models (Cancer Res. 76, 6153–6158 (2016); Cancer Lett. 344, 13–19 (2014)). Therefore, we cannot tell the origin of antitumor effect in this study. The authors are better to use RAG-knockout mice or NSG (NOD/SCID-IL2g-/−) mice model in this study.

Another point to be addressed here is that human CIK cells could be affected by immunosuppressive cells (J Hepatol. 2019 Mar;70(3):449-457). Therefore, we need human immunosuppressive cells in murine HCC models to tell the exact anticancer effect of human CIK cells in vivo system.

>> Thank you very much for these essential recommendations. The mice model is really an important issue for human anti-cancer study. We used the NOD/SCID mice in this study due to the previous well established engraftment mice model for liver cancer [1-5]. In our control group subcutaneously implanted with the J7 HCC cell line (N=5), the tumor grew well and rapidly (Fig. 4B); however, the tumor in the CIK group grew slowly, suggesting that human CIK could help inhibit human tumor growth in immune compromised mice. We think NOD/SCID mice model is reasonable for this study. Indeed, it’s one of our limitations to establish human immunosuppressive tumor environment to tell the exact anticancer effect of CIK cells, especially that human CIK cells could be affected by MDSC.

We added a new paragraph in “Discussion” to describe the reason of our NOD-SCID mice model and the limitation of lack of human immunosuppressive cells in our model, as below:

““The mice model is an important issue for human anti-cancer study. NOD/SCID mice contain impaired T cell, B cell lymphocyte and NK cell function, and also lack macrophage and dendritic cell activity as well as reduced complement activity. Therefore, NOD/SCID mice exhibit a sufficient ability to support engraftment with human cancer tissues including liver cancer [1-5], and with hematopoietic cells [6-8]. It provides a reliable model of anti-cancer therapy screening. Although, many cancers still fail to engraft efficiently in NOD/SCID mice, largely due to remaining NK cell activity and other residual innate immune function. This leakiness may be eliminated in the RAG-1 and RAG-2 mice, or especially the NSG (NOD-SCID-IL2γ^−/−^) mice, produced by the targeted mutation of the IL-2 receptor γ-chain locus in a previously bred NOD-SCID strain, lacking T cells, B cells, macrophages, NK and NKT cells, may also be platforms to overcome the human engraft failure. Beyond the endogenous immune cells, xenograft mouse models affect the tumor growth includes the influences on immune cell infiltration and angiogenesis. The immune cell activation patterns or antigen expression between the mouse and the human immune system were other hurdles. Advances in humanized mouse models are remarkable but are not yet fully established in HCC research [1]. Nevertheless, immunosuppressive cells such as myeloid-derived suppressor cells (MDSC), T-regulatory cells and M2 macrophages could also play an important role in the interplay between a patient’s tumor and the immune system. One previous study showed human CIK cells could be affected by MDSC [9]. Taken together, there are our limitations to establish an artificial environment to define the exact anticancer effect of human CIK cells.””

We hope you can accept our explanations and revision. Thank you again for your kindly consideration.

References:

1.	Brown ZJ, Heinrich B, Greten TF. Mouse models of hepatocellular carcinoma: an overview and highlights for immunotherapy research. Nature reviews Gastroenterology & hepatology. 2018;15(9):536-54.

2.	Chow AK-M, Ng L, Lam CS-C, Wong SK-M, Wan TM-H, Cheng NS-M, et al. The Enhanced metastatic potential of hepatocellular carcinoma (HCC) cells with sorafenib resistance. PloS one. 2013;8(11):e78675.

3.	Xia H, Cao J, Li Q, Lv Y, Jia W, Ren W, et al. Hepatocellular carcinoma-propagating cells are detectable by side population analysis and possess an expression profile reflective of a primitive origin. Scientific reports. 2016;6(1):1-11.

4.	Haraguchi N, Ishii H, Mimori K, Tanaka F, Ohkuma M, Kim HM, et al. CD13 is a therapeutic target in human liver cancer stem cells. The Journal of clinical investigation. 2010;120(9):3326-39.

5.	Wu J, Zhang J, Jiang M, Zhang T, Wang Y, Wang Z, et al. Comparison between NOD/SCID mice and BALB/c mice for patient-derived tumor xenografts model of non-small-cell lung cancer. Cancer Management and Research. 2018;10:6695.

6.	Taussig DC, Miraki-Moud F, Anjos-Afonso F, Pearce DJ, Allen K, Ridler C, et al. Anti-CD38 antibody–mediated clearance of human repopulating cells masks the heterogeneity of leukemia-initiating cells. Blood, The Journal of the American Society of Hematology. 2008;112(3):568-75.

7.	Williams S, Alosco T, Croy B, Bankert RB. The study of human neoplastic disease in severe combined immunodeficient mice. Laboratory animal science. 1993;43(2):139-46.

8.	Hudson W, Li Q, Le C, Kersey J. Xenotransplantation of human lymphoid malignancies is optimized in mice with multiple immunologic defects. Leukemia. 1998;12(12):2029-33.

9.	Yu SJ, Ma C, Heinrich B, Brown ZJ, Sandhu M, Zhang Q, et al. Targeting the crosstalk between cytokine-induced killer cells and myeloid-derived suppressor cells in hepatocellular carcinoma. Journal of hepatology. 2019;70(3):449-57.

**List of amendments to manuscript (Manuscript Number: PONE-D-22-17512R1)**

Reviewer’s comments have been addressed in detail in the “**Point-by-point response to reviewer’s comments**”. Modifications have been marked in red in the revised manuscript and summarized by the following points:

**Discussion:**

P22-23: Add a new paragraph in “Discussion” to describe the reason of our NOD-SCID mice model and the limitation of lack of human immunosuppressive cells in our model.

**References:**

P31-32: Add new references [48-56]. The order of references was modified accordingly.
